# Maxwell A. Pollack Award Presentation and Lecture

**DOI:** 10.1093/geroni/igaa057.3203

**Published:** 2020-12-16

**Authors:** Bob Harootyan

## Abstract

The lecture will feature an address by the2019 Pollack Award recipient, Karen Lincoln, PhD, FGSA of the University of Southern California. The2020 Pollack Award recipient is Karl Pillemer, PhD, FGSA. The Maxwell A. Pollack Award for Productive Aging Award recognizes instances of practice informed by research and analysis, research that has directly improved policy or practice, and distinction in bridging the worlds of research and practice. Supported by The New York Community Trust.

